# Succinate prodrugs in combination with atropine and pralidoxime protect cerebral mitochondrial function in a rodent model of acute organophosphate poisoning

**DOI:** 10.1038/s41598-022-24472-3

**Published:** 2022-11-25

**Authors:** Sarah Piel, Joanna I. Janowska, J. Laurenson Ward, Meagan J. McManus, Joshua S. Jose, Jonathan Starr, Malkah Sheldon, Carly L. Clayman, Eskil Elmér, Magnus J. Hansson, David H. Jang, Michael Karlsson, Johannes K. Ehinger, Todd J. Kilbaugh

**Affiliations:** 1 Resuscitation Science Center of Emphasis, The Children’s Hospital of Philadelphia, 3401 Civic Center Boulevard, Philadelphia, PA 19104 USA; 2 Anesthesiology and Critical Care Medicine, The Children’s Hospital of Philadelphia, Philadelphia, USA; 3 Mitochondrial Medicine, Department of Clinical Sciences Lund, Lund University, Lund, Sweden; 4 Abliva AB, Lund, Sweden; 5 Division of Medical Toxicology, Department of Emergency Medicine, University of Pennsylvania School of Medicine, Philadelphia, USA; 6 Department of Neurosurgery, Rigshospitalet, Copenhagen, Denmark; 7 Otorhinolaryngology, Head and Neck Surgery, Department of Clinical Sciences Lund, Lund University, Skåne University Hospital, Lund, Sweden

**Keywords:** Molecular medicine, Phenotypic screening, Cellular neuroscience, Drug development

## Abstract

Pesticides account for hundreds of millions of cases of acute poisoning worldwide each year, with organophosphates (OPs) being responsible for the majority of all pesticide-related deaths. OPs inhibit the enzyme acetylcholinesterase (AChE), which leads to impairment of the central- and peripheral nervous system. Current standard of care (SOC) alleviates acute neurologic-, cardiovascular- and respiratory symptoms and reduces short term mortality. However, survivors often demonstrate significant neurologic sequelae. This highlights the critical need for further development of adjunctive therapies with novel targets. While the inhibition of AChE is thought to be the main mechanism of injury, mitochondrial dysfunction and resulting metabolic crisis may contribute to the overall toxicity of these agents. We hypothesized that the mitochondrially targeted succinate prodrug NV354 would support mitochondrial function and reduce brain injury during acute intoxication with the OP diisopropylfluorophosphate (DFP). To this end, we developed a rat model of acute DFP intoxication and evaluated the efficacy of NV354 as adjunctive therapy to SOC treatment with atropine and pralidoxime. We demonstrate that NV354, in combination with atropine and pralidoxime therapy, significantly improved cerebral mitochondrial complex IV-linked respiration and reduced signs of brain injury in a rodent model of acute DFP exposure.

## Introduction

Acute pesticide poisoning has become a worldwide health concern, with an estimated 385 million cases of unintentional pesticide poisoning annually. The number of intentional pesticide poisoning cases are even higher, resulting in hundreds of thousands of fatalities each year^1–4^. Anticholinergic compounds, such as organophosphates (OPs), are responsible for the majority of all pesticide-related deaths^5^. OP-based nerve agents pose an additional risk to society due to their potential use as chemical warfare agents^5–8^. OPs are considered highly toxic due to their ability to inhibit the enzyme acetylcholinesterase (AChE), which disrupts normal signal transduction in the nervous system. AChE inhibition causes accumulation of the neurotransmitter acetylcholine, which results in an overstimulation of both muscarinic and nicotinic receptors, leading to neurological impairment^9^. Death from acute OP poisoning is primarily due to respiratory failure, with survivors often demonstrating significant neurologic sequelae^8–10^. Current standard of care (SOC) treatment for acute OP poisoning consists of supportive critical care and anticholinergic drugs, such as muscarinic receptor antagonists (e.g. atropine) and AChE reactivators (e.g. pralidoxime). In some cases benzodiazepines are used as adjuvant medication for OP-induced seizures^5,11^. While SOC treatment may alleviate acute symptoms and reduces short term mortality, survivors are at risk of developing significant cognitive and motor function deficits^5,6,12^. Importantly, research has shown that both the acute and long-term neurotoxic effects of OPs can appear independent of the inhibition of AChE^12,13^. However, there is still a significant gap in our mechanistic understanding of neurologic injury in survivors of acute OP poisoning and a critical need for new adjunctive therapies with novel targets.

While the inhibition of AChE is thought to be the main mechanism of injury, there are other important pathways that may contribute to the overall toxicity of these agents. Mitochondrial dysfunction has been reported as one of the primary non-cholinergic toxic effects of OPs^13^. Mitochondria are most well-known for their role in energy production and reactive oxygen species generation. Not only is the brain the organ with the highest energy demand, it is also dependent on healthy mitochondrial function for the generation and recycling of neurotransmitters. Hence, inadequate mitochondrial function may have severe consequences for cognitive and motor function^14,15^. Multiple studies have shown that a wide range of OPs impair mitochondrial ATP production. Mitochondrial complex I (CI), complex II (CII) and complex IV (CIV)-linked mitochondrial metabolism has been reported to be affected by OP pesticides. Interestingly, the CI- and CIV-linked pathways seem to be impaired to a larger extent than CII-linked pathways^16–20^. We hypothesized that mitochondrially targeted therapies could improve mitochondrial bioenergetic function in OP poisoning.

Our research group has focused on developing mitochondrially targeted therapeutics for metabolic crisis in critical care illnesses and is investigating promising therapies to counteract energy deficiency due to chemically-induced mitochondrial dysfunction. A therapeutic strategy under development is cell-permeable prodrugs of the TCA cycle intermediate succinate, originally developed as treatment for CI-related genetic mitochondrial disorders^21^. We have previously shown in vitro that prodrugs of succinate more readily pass through the cell membrane than regular succinate. Inside the cell, succinate is released and directly metabolized through mitochondrial CII of the oxidative phosphorylation (OXPHOS) system, thereby increasing mitochondrial ATP production^21–25^. Additional studies have demonstrated that succinate prodrugs are able to not only bypass CI-linked dysfunction, but also support impaired mitochondrial respiration due to partial CIV inhibition, despite the site of toxicity being downstream of the target of the succinate prodrugs^21–26^. We hypothesized that co-treatment of SOC treatment with atropine and pralidoxime with a cell-permeable succinate prodrug would support mitochondrial function and reduce brain injury during acute poisoning with the OP diisopropylfluorophosphate (DFP). To this end, we developed a rat model of acute DFP poisoning and evaluated the treatment efficacy of the succinate prodrug NV354 as adjunctive therapy to SOC treatment with atropine and pralidoxime.

## Methods

### Perioperative procedures and hemodynamic monitoring

All procedures were approved by the Institutional Animal Care and Use Committee at the Children’s Hospital of Philadelphia and performed in accordance to the National Institutes of Health Guide for the Care and Use of Laboratory Animals and ARRIVE guidelines. 10-week-old female Sprague Dawley rats (200–300 g) were placed in an induction chamber with 5% isoflurane at a FiO2 of 100% and oxygen flow at 2 L/min. After induction, general anesthesia was maintained with 2–2.5% isoflurane via nose cone. A tracheotomy was performed and oxygen flow rate was set to 0.2 L/min (Harvard Inspira Advanced Safety Ventilator, Harvard Apparatus, MA, USA). Tidal volume (6–7.5 mL/kg) and respiratory rate (60–80 bpm) were titrated to maintain a venous pCO_2_ of 35–55 mmHg. Femoral venous and arterial accesses were placed for blood collection, drug administration and invasive blood pressure monitoring purposes (Transport Pro, GE Medical Systems Information Technologies, Inc, WI, USA). Body temperature was maintained at 36–37 °C throughout the experiment using a heating blanket (T/Pump Professional, Stryker Medical, MI, USA).

### Disease model

**Figure 1:**
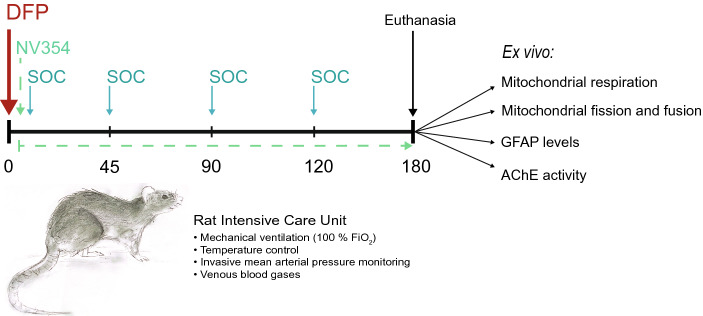
Study design. Once baseline was established, animals received either no treatment (Sham), an acute toxic dose of the organophosphate diisopropylfluorophosphate alone (DFP), DFP and standard of care (SOC) treatment (DFP + SOC), DFP, SOC treatment and the cell-permeable succinate prodrug NV354 (DFP + SOC + NV354) or DFP and treatment with the cell-permeable succinate prodrug NV354 (DFP + NV354). Mean arterial pressure and venous blood gases were measured throughout the experiment. DFP was administered at 3 mg/kg as single, intravenous bolus dose to simulate an acutely toxic one-time exposure. SOC, a combination of atropine (2 mg/kg) and pralidoxime (20 mg/kg), was given as intravenous bolus injections at 5 min following DFP exposure and repeated every 45 min until the end of the experiment. The succinate prodrug NV354 was given immediately after injection of DFP as a single, intravenous bolus dose at 17 mg/kg, followed by continuous venous infusion at 25 mg/kg/h until the end of the experiment. At the end of the experiment (180 min), the animals were euthanized, and brain tissue was immediately collected for ex vivo assessment of mitochondrial respiration, mitochondrial fission and fusion markers, Glial Fibrillary Acidic Protein (GFAP) levels and acetylcholinesterase (AChE) activity.

The organophosphorus compound DFP was selected to simulate acute OP exposure. DFP was originally developed as a potential chemical warfare agent and shows structural and functional similarities to other OP nerve agents, such as sarin, soman, and tabun, but is less toxic, and therefore regularly used in toxicology research^13^. Mean arterial pressure (MAP) and venous blood gases were monitored for 30 min to establish baseline before initiation of exposure and treatment. Following stable baseline, the animals were randomized to the following groups: a) Sham (n = 6), b) DFP alone (DFP, n = 6), c) DFP and SOC treatment (DFP + SOC, n = 6), d) DFP, SOC treatment and the cell-permeable succinate prodrug NV354 (DFP + SOC + NV354, n = 7), and e) DFP and the cell-permeable succinate prodrug NV354 (DFP + NV354, n = 6). Treatment groups and the respective administered agents are depicted in Table 1. Doses of DFP and SOC were selected based on the literature^9,27–29^. DFP was administered at 3 mg/kg as single, intravenous bolus dose to simulate an acutely toxic one-time exposure. Current clinical practice employs repeated dosing of SOC treatment until cholinergic symptoms disappear, and continued monitoring to prevent the reoccurrence of cholinergic symptoms^5^. Due to the limited possibility to monitor cholinergic symptoms in anesthetized rats we standardized SOC drug administration. SOC, a combination of atropine (2 mg/kg) and pralidoxime (20 mg/kg), was given as intravenous bolus injections at 5 min following the start of DFP exposure and repeated every 45 min until the end of the experiment. This treatment regime was considered effective based on the normalization of MAP following administration of SOC treatment (Supplementary Figure S4). In group d) and e), the succinate prodrug NV354 was given immediately after injection of DFP as a single, intravenous bolus dose at 17 mg/kg, followed by continuous venous infusion at 25 mg/kg/h until the end of the experiment. The dosing regimen of NV354 was determined separately and has shown no negative effects on MAP and cerebral mitochondrial respiration (Supplementary Table S4 and Supplementary Fig. S1). The availability of femoral venous access on both sides allowed for administration of SOC boluses and continued infusion of NV354 without any interruption. This study design allowed us to investigate the direct effect of the OP DFP and the treatment effect of NV354 on cerebral metabolism without possible secondary injury due to changes in cardiovascular- and blood–brain–barrier function. Once the respective agent(s) were administered, MAP and venous blood gases were monitored continuously for a total of 180 min, with measurements taken every 10 and 45 min, respectively (i-STAT Handheld Blood Analyzer, Abbott Laboratories, IL, USA; Gem Premier 3000, Instrumentation Laboratory, MA, USA). After 180 min, the animals were euthanized, and blood and brain tissue were immediately extracted. Following tissue collection, the brain was immediately prepared for high-resolution respirometry or snap frozen for assessment of biomarkers (Fig. 1). Blood was processed as described below. Frozen samples were kept at − 80 °C until further analysis.

**Table 1:** Treatment groups and administered agents.

Group	Administered agent		
	DFP	SOC	NV354
(a) Sham	−	−	−
(b) DFP	+	−	−
(c) DFP + SOC	+	+	−
(d) DFP + SOC + NV354	+	+	+
(e) DFP + NV354	+	−	+

*DFP* diisopropylfluorophosphate, *SOC* standard of care (atropine and pralidoxime), *NV354* cell-permeable succinate prodrug.

### Mitochondrial respiration

Mitochondrial function was assessed ex vivo in homogenates of the right frontal cortex. Brain homogenates (1 mg/mL) were prepared fresh as previously described and a respirometric Substrate–Uncoupler–Inhibitor Titration (SUIT) protocol was applied for in-depth characterization of the OXPHOS system and electron transport systems (ETS)^30^. Chemical reagents and concentrations used in the SUIT protocol are provided in Supplementary Table S1. In brief, specific mitochondrially targeted CI-, CII- and CIV-linked substrates were used to assess functionality of the ETS system, the OXPHOS system, and to evaluate the integrity of the inner mitochondrial membrane. For more details on the SUIT protocol see *Jang, et al.*^31^ After completion of the respirometry protocol, the chamber contents were stored at − 80 °C and citrate synthase (CS) activity was measured to correct for any difference in mitochondrial content between samples. CS activity was measured using a commercially available kit according to the manufacturer’s instructions (CS0720; Sigma-Aldrich, St. Louis, MO).

### Western blot

Western blot reagents and antibodies were sourced as stated in Supplementary Table S2 and S3. Brain samples were homogenized in RIPA buffer supplemented with protease and phosphatase inhibitor cocktail (1% v/v) and butylhydroxytoluene (0.05% v/v) at 100 mg/mL using an electric hand-held tissue homogenizer (VWR® Disposable Pellet Mixers and Cordless Motor). Homogenates were then incubated on an orbital shaker for 30 min (Roto-Therm Mini Plus, Benchmark Scientific, Edison, NJ, USA), centrifuged at 16,000 g for 10 min, and the resulting supernatant was subsequently collected. All procedures were performed on ice or at 4 °C. Protein concentrations were measured on the day of the experiment using a Pierce BCA Protein Assay Kit from Thermo Fisher Scientific (Waltham, MA, USA).

Equal amounts of protein (10 µg) were mixed with LDS (1 part LDS:3 parts sample) and 10% (v/v) sample reducing agent, heated at 70 °C for 10 min and loaded on a 4–12% Bis–Tris gel. Proteins were separated by sulfate–polyacrylamide gel electrophoresis (SDS-PAGE) and transferred to nitrocellulose membranes. For assessment of Glial Fibrillary Acidic Protein (GFAP) levels, membranes were blocked overnight at 4 °C on a horizontal shaker in tris-buffered saline and 0.1% tween containing 5% milk powder. The next day, membranes were incubated simultaneously with primary rabbit anti-GFAP antibody (dilution 1:10,000) and primary rabbit anti-glyceraldehyde 3-phosphate dehydrogenase antibody (GAPDH, dilution 1:5000) for 2 h at 4 °C on a horizontal shaker. Subsequently, membranes were washed and incubated with secondary anti-rabbit antibody conjugated to HRP (dilution 1:1000) for 1 h at 4 °C on a horizontal shaker. After a last washing step, membranes were stained with a chemiluminescent substrate reagent kit and relative band density was measured using the iBright CL1000 (Invitrogen by Thermo Fisher Scientific). Antibody incubation of membranes probed for the mitochondrial fission and fusion markers dynamin-related protein 1 (DRP1), mitochondrial fission protein 1 (FIS1), mitofusin 1 and 2 (MFN1, MFN2) and optic atrophy protein 1 (OPA1) was performed in an iBind Western Device (Invitrogen by Thermo Fisher Scientific), with staining and imaging performed as described above. Conditions, antibody dilutions and product information are listed in Supplementary Table S2 and S3. Densitometric analysis was performed using ImageJ version 1.47t.

### Acetylcholine esterase inhibition

A commercially available kit (Acetylcholinesterase Assay Kit, Fluorometric Red, ab138873, Abcam) was used to measure AChE activity in brain and total ChE activity in serum according to the manufacturer’s instructions. Remaining brain homogenates of the sample preparation for high-resolution respirometry were further diluted to a final concentration of 1 mg/mL in the same respiratory buffer (Mir05^30^), briefly sonicated on ice, and centrifuged for 1 min at 13,400 g at 4 °C. The supernatant was collected, and cholinesterase activity was measured in the presence of the butyrylcholinesterase inhibitor tetraisopropyl pyrophosphoramide (500 µM) to exclusively evaluate the inhibition of AChE^27^. Cholinesterase (ChE) activity in blood was measure in serum. To this end, blood was collected just prior to euthanasia and centrifuged at 1300 *g* for 20 min at room temperature. ChE activity in serum was measured undiluted and in the absence of the butyrylcholinesterase inhibitor tetraisopropyl pyrophosphoramide. AChE activity in the brain is expressed as mU/mL whereas ChE activity of serum is expressed as change in fluorescence (Δ RFU).

### Statistics

In a previous unpublished study, we detected a mild yet significant, toxin-induced decrease in cerebral mitochondrial respiration with an effect size of  > 20% with 5–7 animals per group, and hence, a sample size of 6 animals per group was pre-defined for this study. Animals that were unsuccessfully ventilated (pCO_2_ > 70 at any time point) were excluded from the study. Statistical analysis was performed using GraphPad Prism version 9 (GraphPad Software, San Diego, California, USA). Time course analysis of MAP and venous blood gases was performed using two-way ANOVA with Geisser-Greenhouse correction and Dunnett’s multiple comparison test where every time point was compared to baseline (t_0min_). In cases of missing values, a mixed model rather than repeated measure model was applied. Correlation of venous blood gas values with MAP was computed using Spearman’s correlation. For analysis of differences in respiration, AChE and ChE activity, and protein levels, the Shapiro–Wilk test was performed to assess normality of data distribution. Normally distributed data were statistically analyzed using ordinary one-way ANOVA (homogenous variances) or Brown-Forsythe and Welch ANOVA (non-homogenous variances). For comparisons of non-normally distributed data, Kruskal–Wallis test was applied. Tukey’s multiple comparison test with comparison of every group with every other group was used to evaluate treatment effects. Data are presented as median plus interquartile range in brackets ([IQR]), with whiskers indicating minimal and maximal values (box plots). A p value of < 0.05 was considered to indicate significant differences.

## Results

### Mean arterial pressure and blood gas chemistry

**Figure 2:**
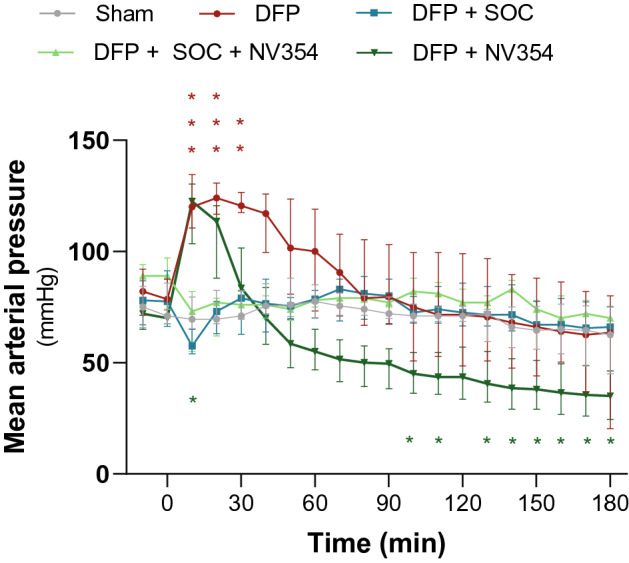
Mean arterial pressure changes. Mean arterial pressure was measured over time in sham animals (Sham) and animals exposed to diisopropylfluorophosphate alone (DFP) with and without standard of care (SOC) treatment and/or the cell-permeable succinate prodrug NV354 (NV354). Time course analysis was performed using two-way ANOVA with Geisser-Greenhouse correction and Dunnett’s multiple comparison test with comparison to baseline (t_0min_). *p < 0.05; **p < 0.01 and p*** < 0.001 indicate significant difference. n = 6–7 per group.

There was variation at baseline of venous blood lactate, bicarbonate, and base excess between groups, which was within normal range for the species^32–34^ and without consistent trends that would indicate pre-exposure respiratory or metabolic acidosis (Supplementary Table S5). Once baseline was established, animals received either no treatment (Sham), DFP alone (DFP), DFP and SOC (DFP + SOC), DFP, SOC and the cell-permeable succinate prodrug NV354 (DFP + SOC + NV354) or DFP and the cell-permeable succinate prodrug NV354 (DFP + NV354) (Table 1). MAP and venous blood gases were continuously measured until the end of the experiment. MAP increased significantly by 42 mmHg ([111–135 mmHg]) compared to baseline (t_0min_) within the first 10 min of exposure to DFP alone (group b, p < 0.001) and stayed significantly elevated for additional 20 min (Fig. 2). 30 min after exposure to DFP the MAP decreased again continuously until the end of the experiment, yet not significantly. DFP exposed animals that were treated with the cell-permeable succinate prodrug NV354 (group e) did also demonstrate a significant increase in MAP from baseline (t_0min_) within the first 10 min after exposure to DFP (p < 0.05), followed by a continuous decrease and significant difference from baseline after 100 min of exposure and treatment (p < 0.05, Fig. 2). SOC treatment alone (group c), or in combination with the cell-permeable succinate prodrug NV354 (group d), resulted in no significant changes in MAP from baseline (t_0min_) (Fig. 2). Venous pCO_2_ remained unchanged in all animals, indicating successful mechanical ventilation (Supplementary Fig. S2a). Blood markers of metabolic acidosis were significantly changed in animals exposed to DFP without SOC treatment (group b and e) (Supplementary Fig. S2). The changes in venous blood pH, lactate, base excess, bicarbonate and SO2c (%) that were observed at the end of the experiment (180 min) demonstrated a strong correlation with MAP (Supplementary Fig. S3), and thus are likely not reflective of systemic metabolic acidosis but rather hypoperfusion. Animals receiving SOC treatment and showing normalized MAP did not present with changes in these markers, further supporting this hypothesis (Supplementary Fig. S2).

### Acetylcholinesterase activity

To confirm OP poisoning, assess the treatment efficacy of pralidoxime, and to exclude that any treatment effects of the succinate prodrug NV354 would be due to the drugs interference with AChE we measured AChE activity in the brain and ChE activity in serum. AChE activity was significantly impaired in the brain of animals exposed to DFP without SOC treatment (Sham vs DFP: p < 0.01, Sham vs DFP + NV354: p < 0.05) (Supplementary Fig. S4a). Surprisingly, SOC treatment alone or in combination with NV354 treatment did not restore AChE activity in the brain (Supplementary Fig. S4a). These results were confirmed by measuring ChE activity in serum of the same animals (Supplementary Fig. S4b).

### Mitochondrial function

**Figure 3:**
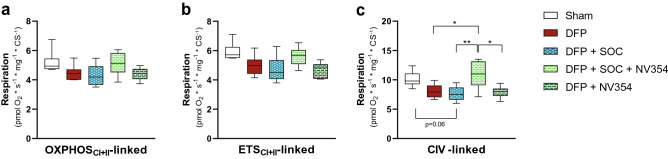
Cerebral mitochondrial respiration. (**a**) Maximal oxidative phosphorylation (OXPHOS) system capacity and (**b**) electron transport system (ETS) capacity supported by mitochondrial complex I and II (CI + II)-linked substrates, as well as (**c**) complex CIV(CIV)-linked mitochondrial respiration supported by the artificial electron donor *N*,*N*,*N*′,*N*′-Tetramethyl-p-phenylenediamine dihydrochloride were measured in brain homogenates of sham animals (Sham), untreated animals exposed to diisopropylfluorophosphate alone (DFP), animals exposed to DFP receiving standard of care (SOC) treatment (DFP + SOC), animals exposed to DFP receiving SOC treatment and the cell-permeable succinate prodrug NV354 (DFP + SOC + NV354) and animals exposed to DFP receiving the cell-permeable succinate prodrug NV354 (DFP + NV354). Data are presented as median plus interquartile range, with whiskers indicating minimal and maximal values. Kruskal–Wallis test or One-Way ANOVA was applied for analysis of differences of non-normally distributed and normally distributed data, respectively, with Tukey’s multiple comparison of every group to every other group. *p < 0.05 and **p < 0.01 indicate significant difference between groups. n = 6 per group.

Mitochondrial function was assessed ex vivo by means of high-resolution respirometry following exposure to DFP with and without SOC and treatment with the cell-permeable succinate prodrug NV354. Brain homogenates of animals exposed to DFP alone showed an overall reduced mitochondrial respiration compared to sham animals (Fig. 3; Supplementary Fig. S5). The use of mitochondrially targeted complex specific substrates for assessment of mitochondrial CI-, CII- and CIV-linked metabolism further revealed a decrease of 19% in CIV-linked mitochondrial respiration in untreated, DFP exposed animals (Sham vs DFP: p = 0.18) (Fig. 3c). The overall decrease in mitochondrial respiration as well as the reduced CIV-linked respiration was not prevented by SOC treatment alone (Sham vs DFP + SOC: p = 0.06; DFP vs DFP + SOC: p = 0.99) (Fig. 3c). Interestingly, the combinational treatment of SOC and the succinate prodrug NV354 preserved CIV-linked respiration (Fig. 3c). CIV-linked respiration was significantly elevated in animals receiving the combinational treatment of SOC and the succinate prodrug NV354 as compared to untreated animals (DFP vs DFP + SOC + NV354: p < 0.05), animals given SOC alone (DFP + SOC vs DFP + SOC + NV354: p < 0.01) but indistinguishable from sham animals (Sham vs DFP + SOC + NV354: p = 0.89). To investigate whether the beneficial effect of the combinational therapy on CIV-linked respiration was due to NV354 or a result of a potential synergistic effect of NV354 and SOC we evaluated the treatment effect of NV354 alone. The decrease in CIV-linked mitochondrial respiration observed in response to DFP was not prevented by NV354 alone (DFP vs DFP + NV354: p = 1.0) (Fig. 3c). Similar beneficial effects of the combinational treatment of SOC and the succinate prodrug NV354 were observed on other respiratory states (Fig. 3, Supplementary Fig. S5). The integrity of the mitochondrial membrane, as determined by LEAK_CI+II_ respiration, was unaffected by DFP or any of the treatment options explored in this study (Supplementary Fig. S5). Moreover, no differences between groups were observed in the mitochondrial fission and fusion markers DRP1, FIS1, MFN1, MFN2 and OPA1 (Supplementary Fig. S6).

### Brain injury

**Figure 4:**
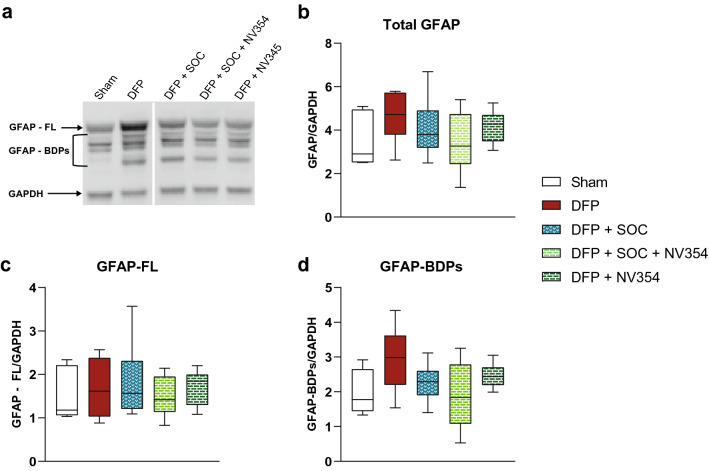
Brain injury markers. Levels of Glial Fibrillary Acidic Protein (GFAP), a biomarker indicative of brain injury, were measured with western blot following exposure to diisopropylfluorophosphate (DFP) with and without standard of care (SOC) treatment and the cell-permeable succinate prodrug NV354 (NV354). (**a**) Representative western blot image to illustrate full-length (FL) GFAP and its Breakdown Products (BDPs). The image was cropped to improve clarity and conciseness. A representative, original and unprocessed image is presented in Supplementary Fig. S7. Quantification of (**b**) total GFAP levels, (**c**) levels of GFAP-FL and (**d**) GFAP-BDPs. Data are presented as median plus interquartile range, with whiskers indicating minimal and maximal values. Kruskal–Wallis test or One-Way ANOVA was applied for analysis of differences of non-normally distributed and normally distributed data, respectively, with Tukey’s multiple comparison test of every group to every other group. n = 6–7 per group.

To evaluate the degree of brain injury following DFP exposure with and without the respective treatment options we measured total levels of the astrocytic, cytoskeletal protein GFAP and its Breakdown Products (BDPs). While there were no statistically significant changes in total GFAP levels or the levels of the full-length (FL) GFAP protein (Fig. 4b, c) we observed a consistent trend towards higher levels of GFAP BDPs when animals were exposed to DFP alone (Fig. 4a, d). Total GFAP BDP levels were 1.7-fold increased by DFP compared to sham animals (Sham: 1.78 [1.44–2.65], DFP: 2.99 [2.2–3.62], Sham vs DFP: p = 0.18) (Fig. 4d). However, when DFP exposed animals received SOC treatment, the succinate prodrug NV354 alone, or both treatments in combination, less GFAP BDPs were detected as compared to untreated animals (DFP + SOC: 2.29 [1.90–2.60], DFP + SOC + NV354: 1.84 [1.08–2.79], DFP + NV354: 2.44 [2.19–2.71]) (Fig. 4a, d). A representative original and unprocessed image of Fig. 4a is shown in Supplementary Fig. S7.

## Discussion

In this study, we demonstrate that mitochondrial CIV function was impaired in the brain during the acute phase of OP poisoning with DFP. This effect was not prevented by SOC treatment with atropine and pralidoxime or treatment with the cell-permeable succinate prodrug NV354 alone. Interestingly, SOC in combination with the cell-permeable succinate prodrug NV354 prevented the OP-induced inhibition of mitochondrial CIV function. Our data indicate that the mitochondrial toxicity of DFP can be mitigated by NV354 as adjunctive therapy to SOC. Moreover, we detected a trend towards increased levels of BDPs of the astrocytic marker GFAP in response to DFP in our model, which is indicative of brain injury. The generation of these BDPs was considerably, yet not significantly, reduced by SOC treatment, treatment with the cell-permeable succinate prodrug NV354, and the combinational therapy of SOC with the cell-permeable succinate prodrug NV354.

Mitochondrial dysfunction is a well-established, non-cholinergic effect of acute poisoning with OPs. Numerous OP-based pesticides have been described to inhibit CI-, CII- and CIV-linked mitochondrial metabolism. Interestingly, the CI- and CIV-linked pathways seem to be impaired to a larger extent than CII-linked pathways^16–20^. To date little is known on the mitochondrial effects of the OP DFP. DFP was originally developed as a potential chemical warfare agent and shows structural and functional similarities to other OP nerve agents, but is less toxic, and therefore regularly used in research on the toxicology of OP-based nerve agents^13^. *Gao et al.*^13^ reported an impairment of mitochondrial axonal transport by DFP that was attributed to changes in mitochondrial transport mechanisms. To our knowledge, this is the first study implicating respiratory chain dysfunction as a non-cholinergic effect of DFP. Despite lack of statistical significance for certain parameters, our data implicate a considerable reduction in mitochondrial respiration by DFP. CIV-linked mitochondrial respiration was reduced by 19%, 24% and 18% in animals exposed to DFP alone (DFP) or in combination with either SOC (DFP + SOC) or NV354 (DFP + NV354), respectively, compared to sham animals (Fig. 3c). It is not unlikely that the actual effect of OPs, such as DFP, on mitochondrial respiration in the brain is more pronounced in patients compared to this animal model due to a longer time between start of exposure and treatment, toxin-induced damage to the blood–brain-barrier and a slowly developing hypotension in response to OPs. An immediate, sudden increase in MAP has been reported to damage blood–brain-barrier function, which would allow not only treatments such as atropine or pralidoxime to reach the brain better but also the toxicant itself^35–38^. At the same time, the slower developing hypotension, which is due to the effect of OPs on muscarinic receptors, can causes reduced cerebral blood flow, which in turn has been reported to impair energy metabolism negatively^39–45^. These secondary factors are likely to aggravate the impairment of mitochondrial function in the clinical setting.

Mitochondrially targeted therapies present a novel approach for the development of adjunctive therapies to SOC^46–48^. The succinate prodrugs were originally developed for primary mitochondrial disease related to CI dysfunction and have been shown to provide electrons to the respiratory chain of the OXPHOS system through CII^21^. The concept is based on the idea that this energy substrate can bypass the dysfunction upstream of CII. Interestingly, *Owiredu, et al.*^26^ recently demonstrated in vitro that a first-generation compound of this novel drug class was also able to increase mitochondrial respiration in peripheral blood cells of patients with acute carbon monoxide poisoning, a known CIV inhibitor. In cases of partial CIV dysfunction, such as during acute DFP or carbon monoxide poisoning, the succinate prodrugs are unable to bypass the dysfunction but instead supply extra reducing equivalents to the respiratory chain via succinate, thereby increasing mitochondrial ATP production despite CIV dysfunction. Following these promising results, it is intriguing to speculate that NV354 may support brain cells energetically during acute OP intoxication and potentially prevents the development of long-term cognitive and motor function deficits.

GFAP is a cytoskeletal astrocytic protein which provides both structural and functional support to the central nervous system (CNS). Astrocytes play an important role in the recovery of CNS injury by maintaining the ionic and molecular compositions of the extracellular fluid, and by promoting neurogenesis^49,50^. They respond to injury by increased proliferation and hypertrophy, resulting in an increase in total GFAP protein^49^. In our model, we were unable to detect significant changes in total GFAP levels in the brain in response to the toxic insult by DFP. This is not surprising, as increased levels of GFAP as sign of astrogliosis have been primarily reported only at later stages following an injury^51,52^. Interestingly, we detected elevated levels of the BDPs of GFAP in untreated animals exposed to DFP. GFAP BDPs between 38 and 44 kDa are a result of pathological activation of the calcium-dependent protease calpain and serve as biomarker for other neurological diseases such as traumatic brain injury^50,53,54^. Under physiological conditions proteolytic activity by calpain is tightly regulated. When intracellular calcium levels are unnaturally elevated, as has been reported for OP exposure, calpain can be falsely activated and initiate excess proteolysis of not only astrocytic cytoskeleton proteins such as GFAP, but also neuronal structural proteins and structural proteins outside of the CNS^4,54–56^. In this study, all three treatment groups trended towards reduced levels of GFAP BDPs. Our findings indicate that GFAP BDPs are a more sensitive marker of early brain injury during acute OP poisoning than total GFAP levels. It remains to be elucidated whether GFAP BDPs are predictive of the long-term development of neurological cognitive and motor function deficits following exposure to OPs.

Our study had certain limitations: First, both treatment strategies, SOC and NV354, were given immediately after start of exposure to DFP. This study design allowed us to investigate the direct effect of DFP and NV354 on cerebral metabolism without possible secondary injury due to prolonged changes in cardiovascular- and related blood–brain–barrier function. Future studies need to investigate delayed treatment regimens to better mimic clinical poisoning and treatment. Second, the time between administration of DFP and the next venous blood gas measurement was 45 min, and as no adjustments to ventilator settings were performed until after this blood gas measurement, the animals were subjected to a transient phase of mild respiratory acidosis. Following venous blood gas measurement, ventilation-settings were adjusted to maintain venous pCO_2_ at 35–55 mmHg. Third, the AChE and ChE activity measurements performed in brain and serum could not confirm the efficacy of pralidoxime in this study. Others have likewise reported a lack of recovery of ChE activity in serum in response to pralidoxime in rats over a comparable exposure time to OPs^28^. In addition, oximes have shown low blood–brain–barrier penetration, and therefore pralidoxime might not have reached the brain during the treatment time of 180 min^57,58^. There is no consensus on the value of oxime therapy as antidote for OP poisoning^5,59,60^. Success of AChE reactivation depends on the OP-AChE binding as well as the oxime used. Because both OP agents and oximes show structural variability, they each have different abilities in binding to AChE and reactivating it. Hence, there is no single oxime that would be effective against all OP pesticides or nerve agents^61–63^. Furthermore, it is well-known that the effectiveness of oxime therapy is dependent on the irreversible aging of the OP-AChE complex, a chemical reaction that can occur within minutes after OPs bind to AChE and which prohibits the reactivation of AChE by oximes^28,60,64^. Taken together, these factors could have contributed to the lack of recovery of AChE/ChE activity in the brain and serum in this study despite pralidoxime treatment. We were nonetheless able to demonstrate the effectiveness of the combined SOC therapy with atropine and pralidoxime. Combinational therapy with both agents counteracted the effect of OPs on cardiovascular function. Our data show that all treatment groups receiving SOC demonstrated a normal, unchanged MAP despite exposure to DFP, while DFP exposed animals not receiving SOC showed alterations in MAP (Fig. 2). To further strengthen this, we plotted MAP before and after administration of SOC for all treatment groups receiving SOC (see Supplementary Fig. S4c). MAP increased significantly from baseline in response to DFP but normalized again after SOC treatment was administered. Whether the stabilization of cardiovascular function was achieved through the actions of atropine alone or the combined effect of atropine and pralidoxime remains to be elucidated in future studies. Lastly, our study would potentially have benefited from a larger sample size (the sample size used was determined based on previous data as described above). Future follow-up studies will use power calculations based on the here presented data for determination of an adequate sample size.

In conclusion, we show that the OP DFP considerably reduced mitochondrial CIV-linked respiration following acute intoxication despite immediate SOC treatment with atropine and pralidoxime, an effect that is possibly more pronounced in patients. Moreover, we demonstrate that SOC treatment in combination with the succinate prodrug NV354 protected cerebral mitochondrial function and reduced signs of brain injury during acute poisoning with the OP DFP. Succinate prodrugs present a novel approach for therapeutic development in this area and show promise as adjunctive therapy to SOC treatment with atropine and pralidoxime. Further studies are needed to investigate the potential of this novel drug class as preventive measure for the development of long-term cognitive- and motor function deficits.

## Supplementary Information

Supplementary Information.

## Data Availability

The datasets used and/or analyzed during the current study are available from the corresponding author on reasonable request.
