# Supplementary material for: Succinate prodrugs in combination with atropine and pralidoxime protect cerebral mitochondrial function in a rodent model of acute organophosphate poisoning
Source: Sci Rep. 2022 Nov 25;12:20329. doi: 10.1038/s41598-022-24472-3 (PMC9700731; doi:10.1038/s41598-022-24472-3)
Supplement: Supplementary file 1 — Supplementary Information. [file 41598_2022_24472_MOESM1_ESM.pdf]

Succinate prodrugs in combination with atropine and pralidoxime protect cerebral mitochondrial function in a rodent model of acute organophosphate poisoning

Supplementary Material

Sarah Piel, PhD[a,b]*, Joanna I. Janowska, MD[a,b], J. Laurenson Ward, BA[a,b], Meagan J. McManus, PhD[a,b], Joshua S. Jose[a,b], Jonathan Starr, BSc[a,b], Malkah Sheldon[a,b], Carly L. Clayman, PhD[a,b], Eskil Elmér, MD, PhD[c,d], Magnus J. Hansson, MD, PhD[c,d], David H. Jang, MD[e], Michael Karlsson, MD, PhD[f], Johannes K. Ehinger, MD, PhD[c,g], Todd J. Kilbaugh, MD[a,b]

[a]Resuscitation Science Center of Emphasis, The Children's Hospital of Philadelphia, Philadelphia, USA; [b]Anesthesiology and Critical Care Medicine, The Children's Hospital of Philadelphia, Philadelphia, USA; [c]Mitochondrial Medicine, Department of Clinical Sciences Lund, Lund University, Lund, Sweden; [d]Abliva AB, Lund, Sweden; [e]Department of Emergency Medicine, Division of Medical Toxicology, University of Pennsylvania School of Medicine, Philadelphia, USA; [f]Department of Neurosurgery, Rigshospitalet, Copenhagen, Denmark; [g]Otorhinolaryngology, Head and Neck Surgery, Department of Clinical Sciences Lund, Lund University, Skåne University Hospital, Lund, Sweden

*Corresponding author: Sarah Piel, PhD, Resuscitation Science Center of Emphasis, The Children's Hospital of Philadelphia, 3401 Civic Center Boulevard, Philadelphia, PA, 19104, USA. E-mail: piels@chop.edu

Keywords: cell-permeable succinate, diispropylfluorophosphate, emergency medicine, mitochondria, organophosphates, toxicity

Supplementary Table S1. Substrate-Uncoupled-Inhibitor Titration (SUIT) Protocol

Reagent	Final concentration
Brain homogenate (mg/mL)	1
Digitonin (µg/mL)	12.5
Malate (mM)	2
Pyruvate (mM)	5
ADP (mM)	3
Glutamate (mM)	5
Succinate (mM)	20
Oligomycin (µg/mL)	1
FCCP (µM per titration step)	0.2
Rotenone (µM)	2
Antimycin A (µg/mL)	1
Ascorbate (mM)	2
TMPD (mM)	0.5
Sodium Azide (mM)	120

Mitochondrial respiration of brain homogenate was evaluated using the Oxygraph-2k (Oroboros Instruments, Innsbruck, Austria) at 37°C, an active chamber volume of 2 mL, a stirrer speed of 750 RPM and measurements every 2 seconds. The SUIT protocol was performed in the below order and the final concentrations as indicated.

Supplementary Table S2. Western blot reagents

Item	Vendor	Catalog number
Pierce™ RIPA Buffer	Thermo Fisher Scientific	89901
Butylhydroxytoluene	Sigma-Aldrich	B1378
Protease and Phosphatase inhibitor cocktail	Sigma-Aldrich	PPC1010
4X Bolt™ LDS Sample Buffer	Thermo Fisher Scientific	B0007
Bolt Sample Reducing Agent	Thermo Fisher Scientific	B0009
NP MES SDS Running Buffer 20X	NC9195866	NP0002
Bolt™ 4 to 12%, Bis-Tris, 1.0 mm, Mini Protein Gel, 15-well	NC9195866	NW04125BOX
Seeblue Plus2 Protein ladder	Thermo Fisher Scientific	LC5925
Iblot™ 2 Transfer Stacks, nitrocellulose	Thermo Fisher Scientific	IB23001
Novex iBind Solution Kit	Fisher Scientific	SLF1020
Novex iBind cards	Thermo Fisher Scientific	SLF1010
Tris buffered saline (10X, with 0.5% Tween-20, pH 7.4)	Fisher Scientific	NC9195866
Milk protein	Lab Scientific bioKEMIX	978-907-4243
SuperSignal™ West Pico PLUS Chemiluminescent Substrate	Thermo Fisher Scientific	34580

Supplementary Table S3. Product information and dilutions of primary and secondary antibodies

Marker	Dilution	Diluent	Vendor	Catalog number
Rabbit anti-DRP1 primary antibody	1:200	iBind solution	Abcam	Ab154879
Rabbit anti-FIS1 primary antibody	1:15000	iBind solution	Thermo Fisher Scientific	50-172-6742
Rabbit anti-MFN1 primary antibody	1:15000	iBind solution	Abcam	Ab221661
Rabbit anti-MFN2 primary antibody	1:2000	iBind solution	Abcam	Ab124773
Rabbit anti-OPA1 primary antibody	1:2000	iBind solution	Abcam	Ab42364
Rabbit anti-GFAP primary antibody	1:10000	Ab5804	Millipore Sigma	Ab5804
Rabbit anti-GAPDH primary antibody	1:5000	iBind solution: DRP1, FIS1, MFN1, MFN2, OPA1<br>TBST + 5 % milk: GFAP	Thermo Fisher Scientific	PIPA585074
Goat anti-Rabbit IgG (H+L) Secondary Antibody, HRP	1:1000	iBind solution: DRP1, FIS1, MFN1, MFN2, OPA1<br>TBST + 5 % milk: GFAP	Thermo Fisher Scientific	A16096

Definition of abbreviations: DRP1 = dynamin-related protein 1; FIS1 = mitochondrial fission 1 protein; MFN1 = mitofusin 1; MFN2 = mitofusin 2; OPA1 = optic atrophy protein 1; GFAP = glial fibrillary acidic protein; GAPDH = glyceraldehyde 3-phosphate dehydrogenase; HRP = Horseradish Peroxidase; TBST = Tris buffered saline with 0.1% Tween, pH 7.4.

Supplementary Table S4. Statistical analysis of the effect of the cell-permeable prodrug of succinate NV354 on mean arterial pressure over time in healthy rats

Group	Time points (min)	Summary	P value	Group	Time points (min)	Summary	P value
Sham	0 vs. -10.00	ns	0.992	NV354	0 vs. -10.00	ns	0.997
	0 vs. 10.00	ns	0.230		0 vs. 10.00	ns	0.921
	0 vs. 20.00	ns	0.957		0 vs. 20.00	ns	0.893
	0 vs. 30	ns	1.000		0 vs. 30	ns	0.868
	0 vs. 40.00	ns	0.882		0 vs. 40.00	ns	0.899
	0 vs. 50.00	ns	0.993		0 vs. 50.00	ns	1.000
	0 vs. 60	ns	0.994		0 vs. 60	ns	1.000
	0 vs. 70.00	ns	1.000		0 vs. 70.00	ns	1.000
	0 vs. 80.00	ns	1.000		0 vs. 80.00	ns	1.000
	0 vs. 90	ns	1.000		0 vs. 90	ns	1.000
	0 vs. 100.00	ns	0.997		0 vs. 100.00	ns	1.000
	0 vs. 110.00	ns	0.973		0 vs. 110.00	ns	1.000
	0 vs. 120	ns	0.938		0 vs. 120	ns	1.000
	0 vs. 130.00	ns	0.505		0 vs. 130.00	ns	1.000
	0 vs. 140.00	ns	0.569		0 vs. 140.00	ns	1.000
	0 vs. 150	ns	0.470		0 vs. 150	ns	0.999
	0 vs. 160.00	ns	0.538		0 vs. 160.00	ns	1.000
	0 vs. 170.00	ns	0.534		0 vs. 170.00	ns	0.999
	0 vs. 180	ns	0.488		0 vs. 180	ns	1.000

Mean arterial pressure was measured over time in sham animals (Sham) and animals treated with the cell-permeable succinate prodrug NV354 (NV354). Time course analysis was performed using two-way ANOVA with Geisser-Greenhouse correction and Dunnett's multiple comparison test where every time point was compared to baseline (t_{0min}). Sham: n=6. NV354: n=3.

Supplementary Table S5. Physiological baseline characteristics

Variable	Sham	DFP	DFP + SOC	DFP + SOC + NV354	DFP + NV354
Weight	270 (258, 297)	267 (257, 275)	266 (259, 296)	265 (237, 283)	247 (237, 263)
Temperature (°C)	35.35 (35.13, 36.28)	36.10 (34.93, 36.73)	37.00 (36.65, 37.93)	36.60 (36.20, 37.60)	37.20 (35.90, 38.28)
MAP (mmHg)	71 (68, 86)	79 (70, 88)	78 (66, 91)	70 (68, 74)	70 (67.50)
pCO_2 (mmHg)	46 (43, 50)	50 (43, 55)	44 (38, 47)	44 (39, 47)	50 (46, 52)
SaO_2 (%)	91 (86, 95)	90 (83, 90)	93 (82, 93)	88 (86, 91)	86 (86, 91)
pH	7.41 (7.39, 7.44)	7.37 (7.35, 7.40)	7.39 (7.38, 7.40)	7.39 (7.34, 7.42)	7.38 (7.35, 7.40)
Glucose (mmol/L)	244 (210, 280)	252 (205, 296)	255 (195, 274)	220 (212, 247)	264 (235, 283)
Lactate (mmol/L)	1.5 (1.3, 2.0)	1.3 (1.2, 1.5)	1.9 (1.6, 2.1)	1.6 (1.5, 2.5)	2.4 (1.7, 2.7) *compared to DFP
Bicarbonate (mmol/L)	30 (27, 32)	29 (26, 31)	26 (23, 28) *compared to Sham	27 (25, 28)	28 (28, 30)
Base Excess (mmol/L)	5.5 (3.0, 7.3)	3.0 (1.5, 5.3)	1.0 (-2.0, 3.5)	1.3 (0.0, 2.0) *compared to Sham	3.0 (2.8, 4.8)
Na^+ (mmol/L)	137 (136, 137)	136 (135, 141)	138 (136, 143)	139 (138, 139)	138 (137, 139)
K^+ (mmol/L)	4.3 (4.1, 4.7)	4.7 (3.9, 5.0)	4.6 (3.6, 4.8)	4.0 (3.8, 4.1)	4.6 (4.3, 5.1)
Ca^{++} (mmol/L)	1.42 (1.38, 1.45)	1.38 (1.20, 1.41)	1.34 (1.26, 1.40)	1.30 (1.28, 1.41)	1.37 (1.29, 1.46)

Baseline characteristics were measured up to 30 min prior to exposure to diisopropylfluorophosphate (DFP) with and without standard of care (SOC) and the cell-permeable succinate prodrug NV354 (NV354). Values are expressed as median plus interquartile range. Kruskal-Wallis test or One-Way ANOVA was applied for analysis of differences of non-normally distributed and normally distributed data, respectively. Tukey's multiple comparison test with comparison of every group to every other group was used to evaluate the treatment efficacy of the succinate prodrug. $p < 0.05$ was considered to indicate significant differences. n= 6-7 per group. *Definition of abbreviations:* MAP = mean arterial pressure.

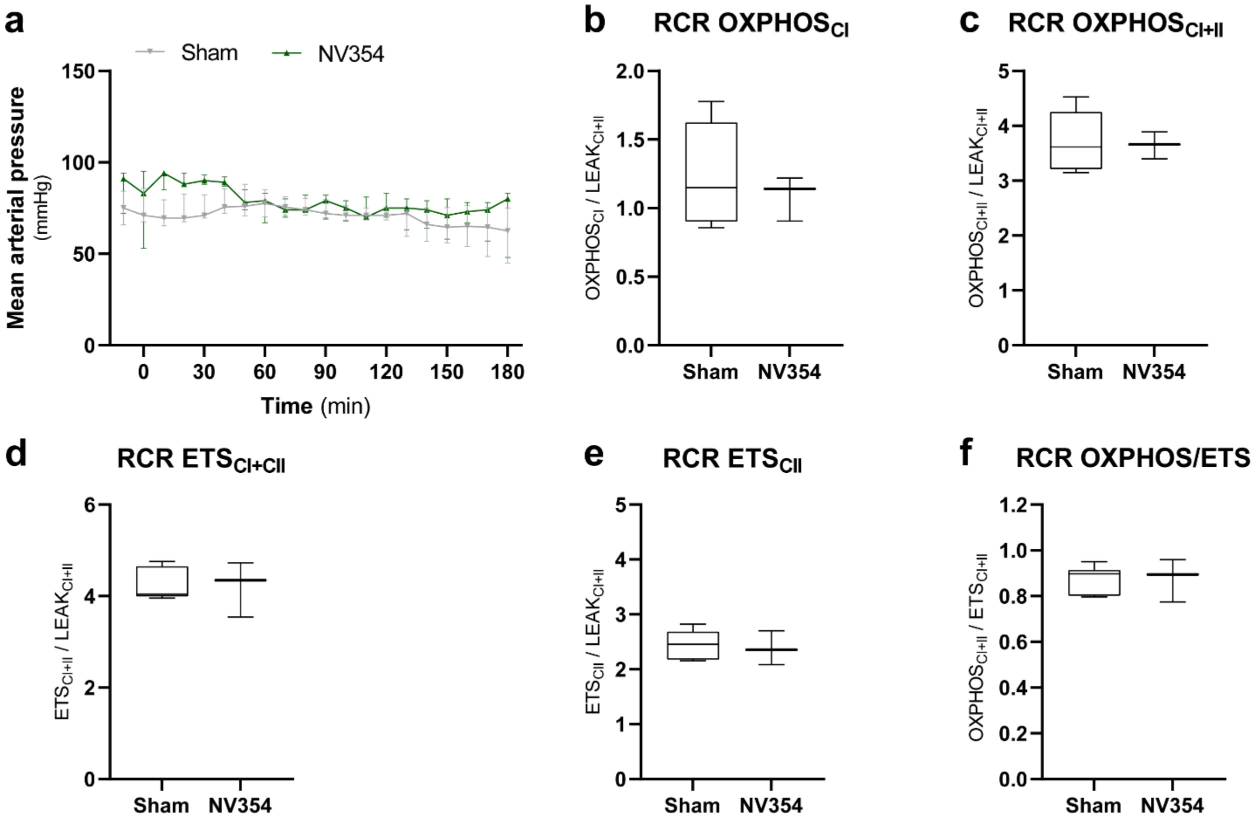

Supplementary Figure S1. Effect of the cell-permeable succinate prodrug NV354 on mean arterial pressure and cerebral mitochondrial respiration of healthy rats. Animals received either no treatment (Sham) or a single, intravenous bolus dose of NV354 at 17 mg/kg, followed by continuous infusion at 25 mg/kg/h over 180 min. (**a**) Mean arterial pressure was measured continuously over 180 min. Mitochondrial respiration of brain homogenates (1 mg/mL) was measured immediately after euthanasia using an Oroboros O2k oxygraphy and a Substrate-Uncoupler-Inhibitor Titration protocol. Respiratory control ratios (RCR) reflective of (**b**) mitochondrial complex I-linked metabolism, (**c-d**) convergent complex I +II -linked metabolism and (**e**) complex II-linked metabolism were evaluated. (**f)** Additionally, the phosphorylation system control ratio (OXPHOS/ETS) was calculated to inform about any limitation of oxidative phosphorylation by CV-linked metabolism. Data are expressed as median plus interquartile range, with whiskers indicating minimal and maximal values. Time course analysis was performed using two-way ANOVA with Geisser-Greenhouse correction and Dunnett's multiple comparison test and comparison to baseline (t_{0min}). Respiratory control ratios were statistically compared using a t-test. $p < 0.05$ was considered to indicate significant differences. Sham: n=6. NV354: n=3. *Definition of abbreviations:* OXPHOS$_{CI}$ = oxidative phosphorylation capacity linked to complex I metabolism; OXPHOS$_{CI+II}$ = oxidative phosphorylation capacity linked to complex I and II metabolism; LEAK$_{CI+II}$ = mitochondrial respiration dependent on the leakiness of the mitochondrial membrane in the presence of complex I and II linked substrates; ETS$_{CI+II}$ = electron transport system capacity linked to complex I+II metabolism; ETS$_{CII}$ = electron transport system capacity linked to complex II metabolism; OXPHOS$_{CI+II}$/ETS$_{CI+II}$ = phosphorylation system control ratio.

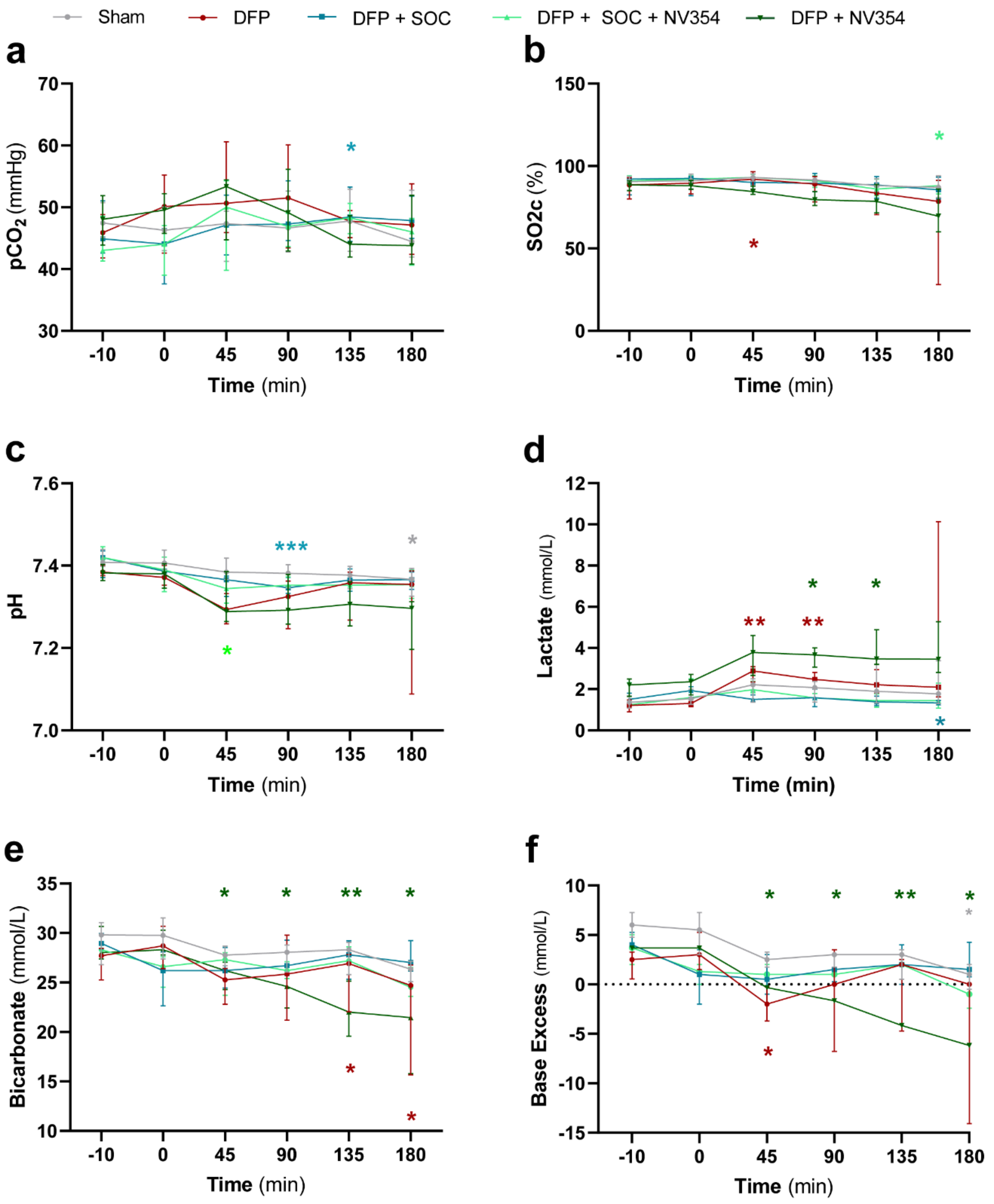

Supplementary Figure S2. Venous blood gases. Venous blood gases were measured continuously over 180 min in sham animals (Sham) and animals exposed to diisopropylfluorophosphate alone (DFP), DFP with standard of care (SOC) treatment (DFP + SOC), DFP with SOC and the cell-permeable succinate prodrug NV354 (DFP + SOC + NV354) and DFP with the cell-permeable succinate prodrug NV354 (DFP + NV354). Venous (**a**) pCO_2, (**b**) oxygen saturation (SO2c, %), (**c**) pH, (**d**) lactate (mmol/L), (**e**) bicarbonate (mmol/L) and (**f**) base excess (mmol/L) are presented as median plus interquartile range. Two-way ANOVA with Geisser-Greenhouse correction and Dunnett's multiple comparison test was performed. *: $p<0.05$, **: $p<0.01$ and p: ***<0.001 indicate significant difference to baseline (t_{0min}). n=6-7 per group.

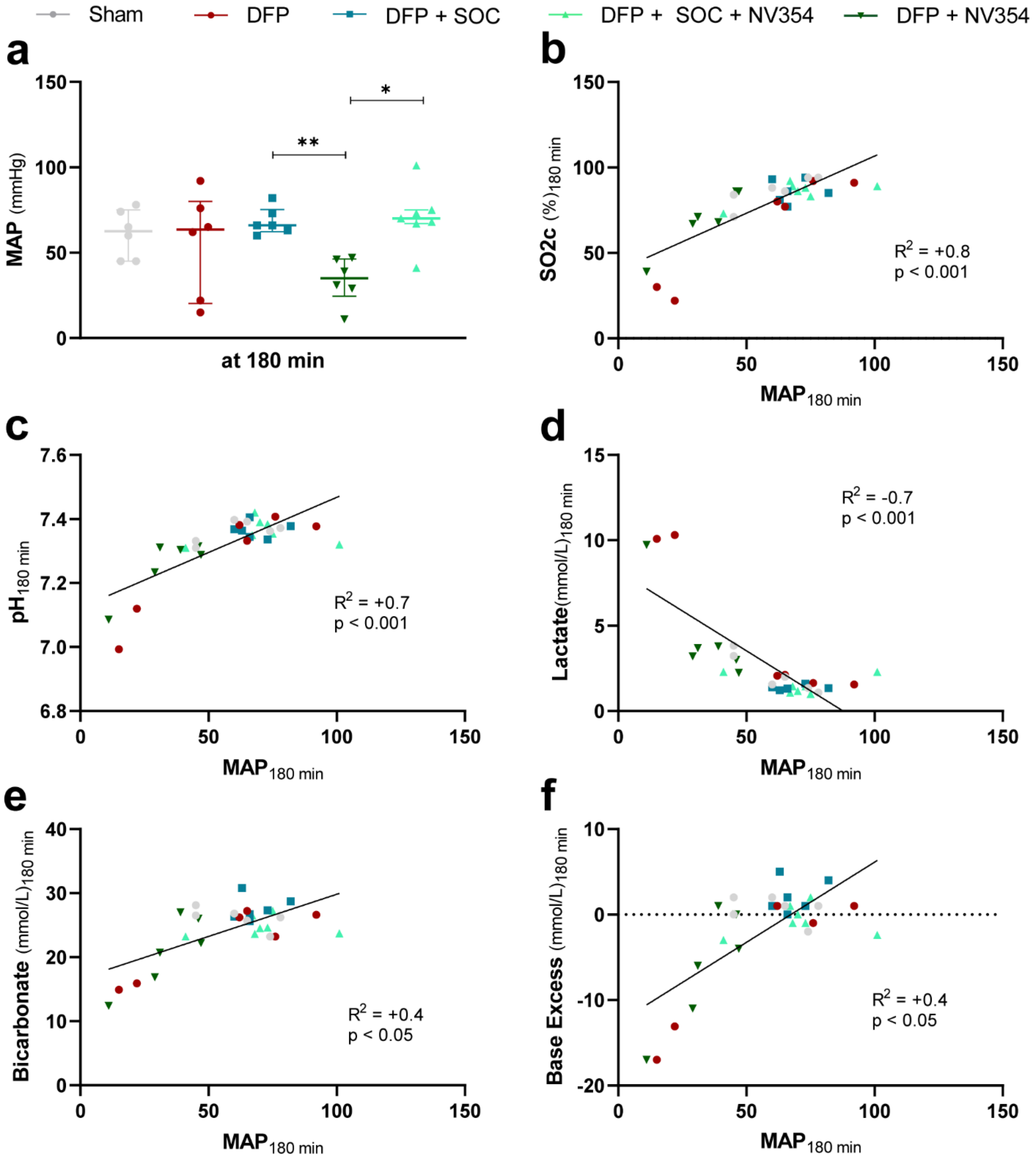

Supplementary Figure S3. Correlation of venous blood gas values with mean arterial pressure. (**a**) Mean arterial pressure (MAP) and (**b-f**) venous blood gases were measured at the end of exposure (180 min) to diisopropylfluorophosphate (DFP) with and without standard of care (SOC) treatment and treatment with the cell-permeable succinate prodrug NV354. Venous blood (**b**) oxygen saturation (SO2c, %), (**c**) pH, (**d**) lactate (mmol/L), (**e**) bicarbonate (mmol/L) and (**f**) base excess (mmol/L) at the end of exposure (180 min) were correlated with MAP at the end of exposure (180 min). For correlation analysis venous blood gases were plotted over MAP independent of their groups. Correlation was evaluated using Spearman's correlation coefficient. A p value of < 0.05 was considered to indicate significant correlation and a correlation coefficient (R^2) of 0.1-0.3 indicated a small correlation, 0.3-0.5 indicated a moderate correlation and 0.5-0.8 indicated a strong correlation. n=31.

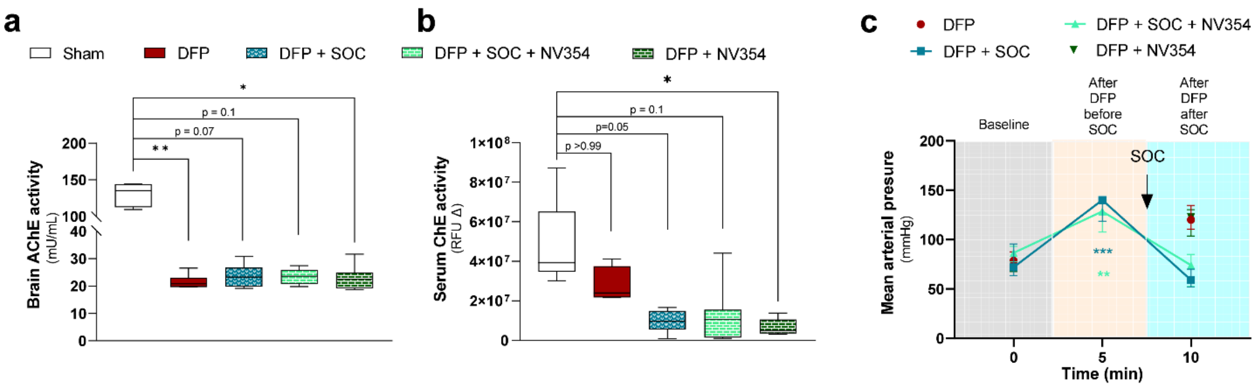

Supplementary Figure S4. Cholinergic effects and mean arterial pressure changes. Following exposure to diisopropylfluorophosphate (DFP) with and without standard of care treatment (SOC) and the cell-permeable succinate prodrug NV354 (NV354) animals were euthanized, and tissues were collected for assessment of (**a**) acetylcholinesterase activity (AChE) in brain and (**b**) cholinesterase (ChE) activity in serum. (**c**) Mean arterial pressure was measured at baseline (t_{0min}), before administration of SOC ($t_{5\ min}$) and after administration of SOC (t_{10min}) in animals exposed to DFP and receiving SOC treatment. No mean arterial pressure was recorded at 5 min in the groups receiving no SOC treatment (DFP) or NV354 alone (DFP + NV354). Data are presented as median plus interquartile ranges, with whiskers indicating minimal and maximal values (box plots). Time course analysis was performed using two-way ANOVA with Geisser-Greenhouse correction and Dunnett's multiple comparison test with comparison to baseline (t_{0min}). Kruskal-Wallis test was applied for analysis of differences of AChE and ChE activity. *: p<0.05, **: p<0.01 and p: ***<0.001 indicate significant difference. n = 4-7 per group.

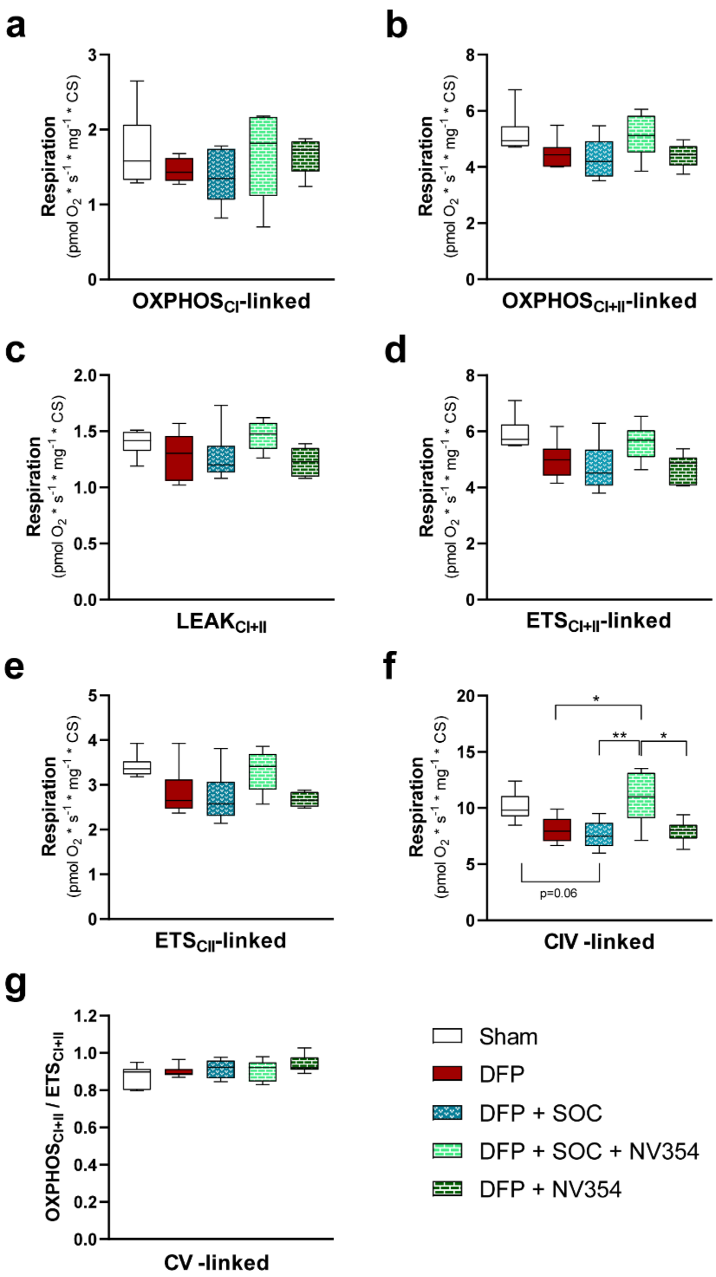

Supplementary Figure S5. Cerebral mitochondrial respiration. Mitochondrial respiration of brain homogenates (1 mg/mL) was measured using an Oroboros O2k oxygraph following exposure to diisopropylfluorophosphate (DFP) with and without standard of care (SOC) treatment and the cell-permeable succinate prodrug NV354 (NV354). Data are presented as median plus interquartile range with whiskers indicating minimal and maximal values. Kruskal-Wallis test or One-Way ANOVA was applied for analysis of differences of non-normally distributed and normally distributed data, respectively, with Tukey's multiple comparison of every group to every other group. *: $p<0.05$ and **: $p<0.01$ indicate significant difference between groups. n=6-7. (**a**) OXPHOS$_{CI}$ = complex I-linked oxidative phosphorylation capacity, (**b**) OXPHOS$_{CI+II}$ = complex I and II-linked oxidative phosphorylation capacity, (**c**) LEAK$_{CI+II}$ = complex I and II-linked mitochondrial oxygen consumption dependent on the leakiness of the mitochondrial membrane, (**d**) ETS$_{CI+II}$ = complex I and II-linked electron transport system capacity, (**e**) ETS$_{CII}$ = complex II-linked electron transport system capacity, (**f**) CIV = complex IV-linked respiration and (**g**) OXPHOS$_{CI+II}$/ETS$_{CI+II}$ = phosphorylation system control ratio are shown.

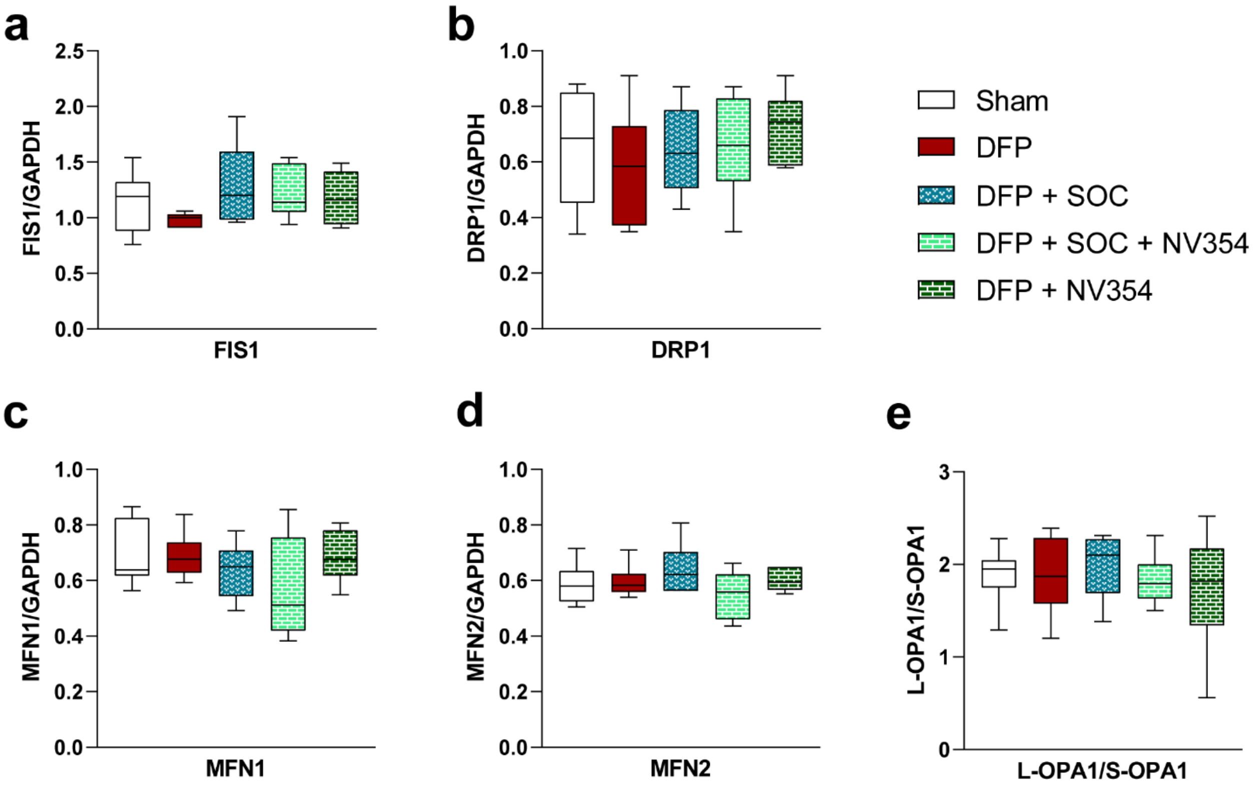

Supplementary Figure S6. Cerebral mitochondrial fission and fusion markers. Markers of mitochondrial fission and fusion were assessed following exposure to diisopropylfluorophosphate (DFP) with and without standard of care (SOC) treatment and the cell-permeable succinate prodrug NV354 (NV354). The mitochondrial fission markers (**a**) FIS1 and (**b**) DRP1, as well as the fusion markers (**c**) MFN1 and (**d**) MFN2 were evaluated. In addition, (**e**) the ratio of the long isoform of OPA1 over the short isoform of OPA1 (L-OPA1/S-OPA1) was calculated. There was no statistical difference found between groups. Data are presented as median plus interquartile range with whiskers indicating minimal and maximal values. Kruskal-Wallis test or One-Way ANOVA was applied for analysis of differences of non-normally distributed and normally distributed data, respectively, with Tukey's multiple comparison of every group to every other group. n=6-7. *Definition of abbreviations*: DRP1 = dynamin-related protein 1; FIS1 = mitochondrial fission 1 protein 1; MFN1 = mitofusin 1; MFN2 = mitofusin 2; OPA1 = optic atrophy protein 1; GAPDH = glyceraldehyde 3-phosphate dehydrogenase.

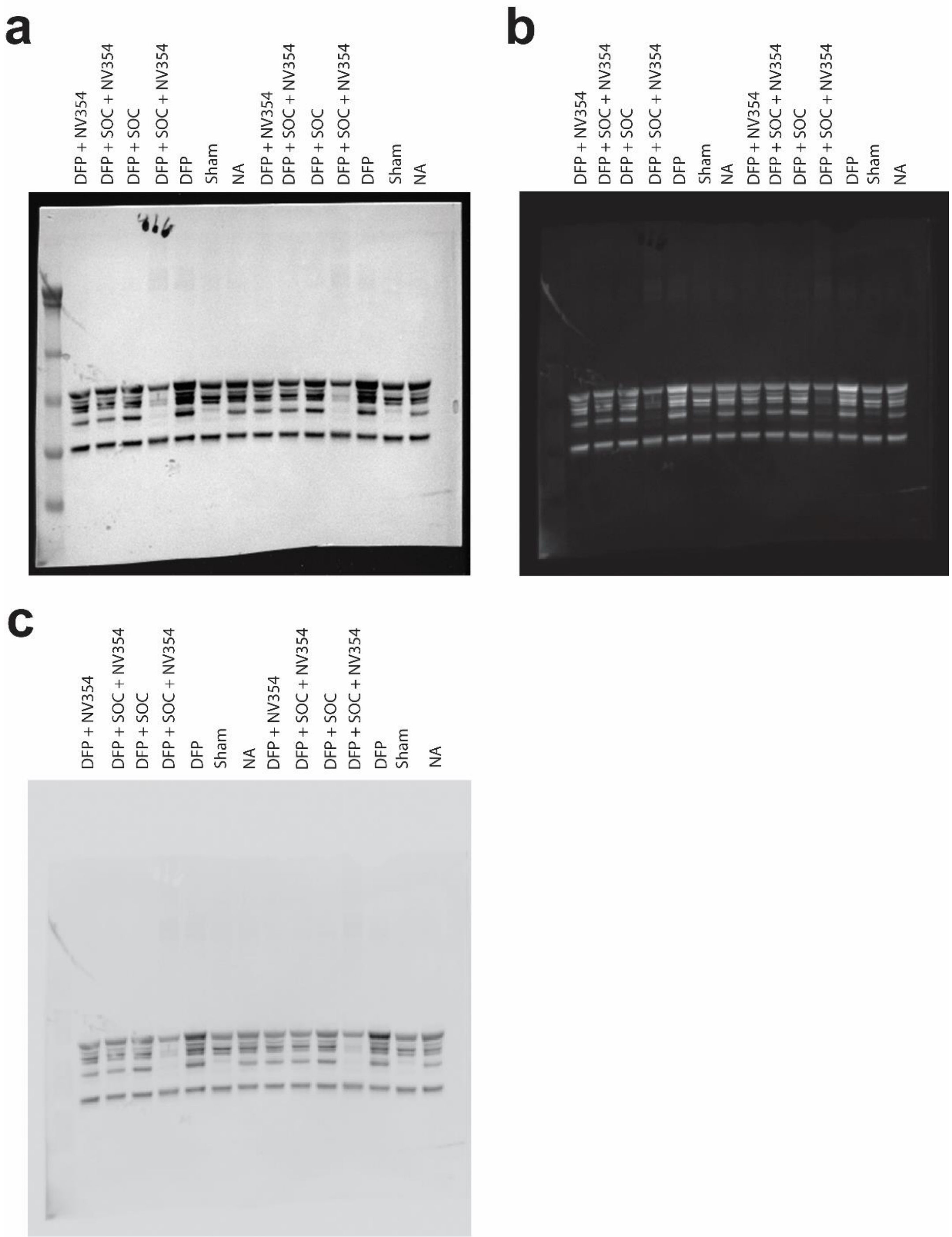

Supplementary Figure S7. Brain injury markers. Levels of Glial Fibrillary Acidic Protein (GFAP), a biomarker indicative of brain injury, were measured by Western blot following exposure to diisopropylfluorophosphate (DFP) with and without standard of care (SOC) treatment and the cell-permeable succinate prodrug NV354 (NV354). (**a**) The membrane overlay of the full-length, original, unprocessed imaged of Figure 4a is shown. The membrane overlay allows to see the pre-stained protein ladder, which aids in identifying molecular weights of the respective markers. (**b**) Raw chemiluminescent image. (**c**) Inverted raw chemiluminescent image used for densitometric analysis with ImageJ version 1.47t. Sample NA is outside of the scope of the present manuscript and therefore not included in the analysis.
